# The Use of Rare Earth Glass Microspheres and Graphene Quantum Dots Glass Microspheres for Biological Applications: Cancer Insight

**DOI:** 10.34172/apb.025.45113

**Published:** 2025-07-20

**Authors:** Jéssica Ingrid Faria De Souza, Natália Cristina Gomes-da-Silva, Filipe Ferreira Ascenção, Beatriz Da Silva Batista, Luciana Magalhães Rebelo Alencar, Pierre Basílio Almeida Fechine, Eduardo Ricci-Junior, Ralph Santos-Oliveira

**Affiliations:** ^1^Brazilian Nuclear Energy Commission, Nuclear Engineering Institute, Laboratory of Nanoradiopharmacy and Synthesis of New Radiopharmaceuticals, Rio De Janeiro, 21941906, RJ, Brazil; ^2^Department of Physics, Federal University of Maranhão, São Luis, 65065690, MA, Brazil; ^3^Grupo De Química De Materiais Avançados (GQMat), Departamento De Química Analítica e Físico-Química, Universidade Federal Do Ceará–UFC, Campus Do Pici, CP 12100, Fortaleza, Ceará, CEP 60451-970, Brazil; ^4^School of Pharmacy (Faculty), Federal University of Rio de Janeiro, Rio de Janeiro Brazil; ^5^Rio De Janeiro State University, Laboratory of Radiopharmacy and Nanoradiopharmaceuticals, Rio De Janeiro, 23070200, RJ, Brazil

**Keywords:** Oncology, Therapy, Prostate cancer, Breast cancer

## Abstract

**Purpose:**

This study explores the use of glass microspheres doped with rare earth elements, specifically samarium (Sm) and neodymium (Nd), and graphene quantum dots (GQDs) in biological applications, particularly cancer therapy.

**Methods:**

Glass microspheres were synthesized using an eco-friendly approach with recycled glass and subsequently doped with Sm, Nd, or GQDs. The samples were characterized by scanning electron microscopy (SEM) and energy-dispersive X-ray spectroscopy (EDS). In vitro cytotoxicity was assessed in MCF-7 (breast cancer) and DU-145 (prostate cancer) cell lines.

**Results:**

In vitro assays demonstrated that these doped microspheres significantly reduced cell viability in breast (MCF-7) and prostate (DU-145) cancer cell lines. The GQD microspheres showed a marked reduction in cell proliferation, attributed to mechanisms involving apoptosis and reactive oxygen species (ROS) production. Sm and Nd microspheres also decreased cell survival, with Nd microspheres showing the highest efficacy.

**Conclusion:**

The study highlights the potential of rare earth elements and GQDs in developing advanced nanotherapeutic agents for cancer treatment, emphasizing their role in disrupting cellular functions and promoting cytotoxic effects in tumor cells.

## Introduction

 Glass microspheres are small, spherical particles made from glass, typically ranging from a few micrometers to several millimeters in diameter. These microspheres are characterized by their uniform size, smooth surface, and chemical inertness, making them versatile in a wide range of applications, including healthcare.^1-4^

 In medical assistance, glass microspheres are utilized for both therapeutic and diagnostic purposes. They are employed as carriers for drug delivery, where their surface can be modified to attach to specific molecules, enabling targeted delivery to specific tissues or cells. This targeted approach minimizes systemic side effects and enhances the efficacy of treatments, particularly in chemotherapy and radiotherapy.^5-7^

 Moreover, glass microspheres are used in medical imaging as contrast agents. Their unique optical properties allow them to enhance the contrast in imaging techniques like ultrasound, magnetic resonance imaging (MRI), and computed tomography (CT) scans, facilitating better visualization of tissues and organs. This is crucial for accurate diagnosis and monitoring of various medical conditions.^8-11^

 Another innovative application of glass microspheres is in tissue engineering. They serve as scaffolds or support structures in the regeneration of bone and soft tissues.^12^ The biocompatibility of glass microspheres ensures that they integrate well with biological tissues, supporting cell growth and tissue repair.

 Rare earth microspheres (REMs) are a class of nanomaterials derived from rare earth elements (REEs), which include the fifteen lanthanides, scandium, and yttrium. These elements possess unique electronic, magnetic, and optical properties, making them highly valuable in various technological and scientific applications. REMs harness these properties at the nanoscale, offering enhanced performance and novel functionalities compared to their bulk counterparts. They are emerging as promising tools in cancer research and therapy due to their unique physicochemical properties. Their applications range from diagnostics to therapeutics, and they are leveraging their luminescent, magnetic, and catalytic characteristics to significantly improve cancer detection and treatment efficacy.^13^

 Samarium (Sm) is a REE that has garnered significant attention in nanotechnology and oncology due to its unique magnetic, electronic, and radiative properties. Samarium-based micro/nanoparticles (SmNPs) offer diverse applications, particularly in diagnostic imaging, targeted therapy, and radiotherapy enhancement.^14^

 Neodymium (Nd), a member of the lanthanide series, is renowned for its magnetic properties and applications in various technological fields. Neodymium-based micro/nanoparticles (NdNPs) have recently gained attention in nanotechnology and oncology due to their unique optical, magnetic, and catalytic properties.^15^

 Graphene quantum dots (GQDs) are a novel class of carbon-based nanomaterials characterized by their small size, typically ranging from 2 to 20 nm, and their unique electronic and optical properties. These properties arise from quantum confinement effects, where the electronic and optical behaviors are influenced by the size and shape of the quantum dots, leading to discrete energy levels and size-dependent fluorescence. QQDs are known for their strong and tunable fluorescence, which can be adjusted by modifying their size, shape, and surface chemistry. This property is particularly useful in bioimaging, where GQDs can be used as fluorescent markers to visualize cells and tissues with high resolution. Unlike many conventional quantum dots, GQDs are generally considered to be biocompatible and have low cytotoxicity. This makes them suitable for in vivo applications, including imaging, drug delivery, and biosensing. Also, GQDs have a high surface area to volume ratio, allowing for extensive functionalization with various chemical groups. This property enables the conjugation of biomolecules, such as antibodies, peptides, or drugs, enhancing their specificity and functionality in biological systems. Finally, GQDs are chemically stable, resistant to photobleaching, and can maintain their properties under various physiological conditions, which is critical for reliable long-term applications in medical diagnostics and therapy.^16-19^

 The strong fluorescence of GQDs makes them ideal for bioimaging applications, including fluorescence microscopy, MRI, and CT imaging.^20,21^ They provide high-contrast images and can be used to track biological processes at the cellular and molecular levels. Also, GQDs can be engineered to carry therapeutic agents, enabling targeted drug delivery to specific cells or tissues. This targeted approach reduces systemic toxicity and enhances the therapeutic efficacy of drugs, especially in cancer therapy.

 Breast and prostate cancer are two of the most prevalent cancers worldwide, significantly impacting public health and healthcare systems. Both cancers have distinct epidemiological characteristics, diagnostic challenges, and treatment approaches, contributing to substantial morbidity, mortality, and economic burden.^22,23^ Breast cancer is the most common cancer among women globally, accounting for approximately 24.5% of all new cancer cases in women. In 2020, there were an estimated 2.3 million new cases of breast cancer worldwide, leading to approximately 685,000 deaths in 2020.^24^ The major risk factors include age, family history, genetic mutations (e.g., BRCA1 and BRCA2), hormonal factors, lifestyle factors (e.g., obesity, alcohol consumption), and reproductive history.^25^ Early detection and improved treatment options have increased the 5-year survival rate for localized breast cancer to about 90%. However, survival rates drop significantly for metastatic breast cancer.^26^

 In the United States alone, the annual direct medical cost of breast cancer is estimated to be over $20 billion, including costs for screening, treatment, and follow-up care. Indirect costs, such as lost productivity due to illness and premature death, add significantly to the economic burden, with estimated costs exceeding $10 billion annually.^27,28^

 Prostate cancer is the second most common cancer in men worldwide, accounting for approximately 14.1% of all new cancer cases in men.^29^ In 2020, there were an estimated 1.4 million new cases of prostate cancer globally, leading to approximately 375,000 deaths in 2020. The major risk factors include age, family history, genetic factors (e.g., BRCA2 mutations), race (higher incidence in African American men), and lifestyle factors. The 5-year survival rate for localized prostate cancer is nearly 100%.^30^ However, for advanced or metastatic prostate cancer, the 5-year survival rate drops to about 30%. The annual direct medical cost of prostate cancer in the United States is estimated to be around $10 billion, encompassing screening, treatment, and follow-up care. Indirect costs, including lost productivity due to illness and premature death, add to the economic burden, with estimated costs exceeding $5 billion annually.^31,32^

 Breast and prostate cancer together represent a significant proportion of the global cancer burden. In 2020, they accounted for approximately 14% of all new cancer cases and 10% of all cancer deaths worldwide. The combined morbidity and mortality associated with these cancers highlight the critical need for effective screening, early detection, and advanced treatment strategies. In terms of economic impact, the combined economic burden of breast and prostate cancer is substantial, with direct healthcare costs and indirect costs related to lost productivity and premature death exceeding $35 billion annually in the United States alone.^33-35^

 The development of new drugs for breast and prostate cancer is crucial for several reasons, including addressing unmet clinical needs, improving patient outcomes, and managing the evolving landscape of cancer biology.^36,37^ Despite advances in early detection and treatment, significant challenges that necessitate continued pharmacotherapy innovation remain. Many patients with breast cancer, especially those with advanced or metastatic disease, eventually develop resistance to standard treatments such as hormone therapy (e.g., tamoxifen, aromatase inhibitors), HER2-targeted therapy (e.g., trastuzumab), and chemotherapy. This resistance often leads to disease progression and limited treatment options. Similarly, castration-resistant prostate cancer (CRPC) represents a significant therapeutic challenge. Patients who no longer respond to androgen deprivation therapy (ADT) require new therapeutic options to manage their disease effectively.^38-41^

 Nanotechnology is revolutionizing the field of oncology, particularly in the diagnosis, treatment, and management of breast and prostate cancers. By manipulating materials at the nanoscale, researchers and clinicians can develop more precise, effective, and less toxic interventions for cancer patients.^42-45^ Using nanotechnology to encapsulate chemotherapeutic drugs ensures they are delivered specifically to cancer cells while minimizing systemic toxicity.^46-48^ Targeting ligands, such as antibodies or peptides, can be attached to the surface of these nanoparticles to recognize and bind to specific receptors on cancer cells.^49^

 The necessity for new drugs in breast and prostate cancer is driven by the need to overcome resistance to existing treatments, address tumor heterogeneity, manage metastatic disease, and improve patient outcomes.^50,51^ Advances in targeted therapies, immunotherapy, and precision medicine hold significant promise in transforming the treatment landscape for these cancers. Continued research and development efforts are essential to bring innovative and effective treatments to patients, ultimately reducing the burden of breast and prostate cancer on individuals and healthcare systems.^52-54^

 In this direction, developing new nanodrugs based on rare earth metals and GQDs can represent an important achievement with good results, especially in reducing toxicological aspects related to rare earth use in biological systems. Also, the use of GQDs immobilized in a different platform for cancer therapy can also represent an important achievement. Thus, in this study, we have produced, fully characterized, and *in vitro* evaluated two nanoparticles based on rare earth metals, including samarium (Sm) and neodymium (Nd) and one based on GQDs.

## Materials and Methods

###  Reagents

 All reagents and solvents used in this study were purchased from Sigma-Aldrich (Brazil).

###  Graphene quantum dots production

 The method for producing GQDs dispersions was adapted from a previously published study.^55^ In this procedure, a graphite rod served as the anode, and a platinum wire as the cathode. The electrolyte solution was prepared by combining 63.5 mL of 0.2 M citric acid with 36.5 mL of 0.2 M sodium citrate, yielding a total volume of 100 mL. The electrochemical synthesis was carried out at a constant current of 190 mA for 24 hours using an ICEL PS-1500 adjustable power supply. Following electrolysis, the resulting dispersion was filtered to remove larger particles. The filtered suspension was then concentrated by drying at 60°C, reducing the volume to 10 mL. Subsequently, 50 mL of ethanol was added, and the upper phase, containing purified GQDs, was collected. The purified GQDs were further dried at 60 °C until needed.

 All characterization assays were conducted using a range of techniques, including dynamic light scattering (DLS), Raman spectroscopy, atomic force microscopy (AFM), and powder X-ray diffraction (PXRD). These data, which have been previously published, confirmed the successful production of GQDs.

###  Glass source

 The glass used was from a recycling industry in Rio de Janeiro. The glass composotion was: 75 percent silica, 10 percent lime, and 15 percent soda.

###  Pre-treatment of the glass

 All glasses used in this study was previously washed with a detergent solution and dried at 150ºC for 24h.

###  Glass microsphere doped with Sm, Nd and GQDs

 The production process is protected by the patent BR 10 2023 023825-4. Briefly, recycled glass was used as the primary raw material. This glass was pulverized using a mortar and pestle. A total mass of 20g of the pulverized glass was weighed, and surfactant was added along with 2g of the REEs, i.e., GQDs, samarium oxide and neodymium oxide, respectively. The mixture was then mixed vigorously and heated at 1200 °C for 2 hours. After that, the mixture was cooled to room temperature and pulverized again using a mortar and pestle. The resulting powder was washed twice with distilled water and dried at 100 °C for 24 hours.

###  Particle size

 The powder of the synthesized samples was dispersed in acetone and after drying, they were analyzed in an optical microscope (Olen) with an attached camera. The diameter of the microspheres was measured using Gwyddion software. A sample mean and standard error were obtained from the data set (n = 30).

## Scanning electron microscopy and energy-dispersive X-ray spectroscopy

 The morphology of the microspheres was analyzed using scanning electron microscopy (SEM). SEM measurements were carried out with a scanning electron microscope (Zeiss, Evo) on samples deposited on carbon tapes using a secondary electron detector (SE). The images were obtained with magnifications of up to 20Kx. energy dispersive X-ray spectroscopy (EDS) (Bruker, XFlash 410 M) identified the distribution of chemical elements present in the samples.

###  Statistical analysis 

 The data obtained from the cell viability assay was plotted in the GraphPad Prism 8.1 program. The experiments were carried out at least three times with six experimental replicates. The data was analyzed by one-way ANOVA to determine the difference between the groups and the control. The asterisks show statistical significance. * P < 0.05 was considered significant, ** P < 0.01 was considered highly significant, and ***P < 0.001 was considered very highly significant.

###  Cell lines

####  Breast cancer cells

 MCF-7 breast cancer tumor cells were selected for the study. The cells were obtained from the Rio de Janeiro Cell Bank. The cell lines were cultured in RPM1-1640 medium supplemented with 10% (v/v) fetal bovine serum, 1% (v/v) penicillin/streptomycin antibiotic, and 5 mM glutamine. The cells were grown in a wet oven with 5% carbon dioxide at 37 ºC.

####  Prostate cancer cells

 DU-145 prostate cancer cells were selected for this study and obtained from the Rio de Janeiro Cell Bank. The cells were cultured in RPMI-1640 medium, supplemented with 10% (v/v) fetal bovine serum, 1% (v/v) Penicillin/Streptomycin antibiotic, and 5 mM glutamine. They were maintained in a humidified incubator with 5% carbon dioxide at a constant temperature of 37 ºC.

####  Cell culture

 The cells were expanded into 75 cm^2^ bottles (T75 Corning®). After reaching the ideal confluence of 80%, they were treated with a trypsin solution (0.1%) plus ethylenediaminetetraacetic acid (EDTA) (0.01%) for replating in flat-bottomed transparent 96-well plates. The cells were then plated at 1x10^-4^ cells per well for subsequent cell viability experiments.

###  Treatment with rare earth microspheres and GQDs

 After 24 hours of cell growth on the plates, both tumor cell lines were treated with the rare earth metal microspheres and the GQD microspheres. Each nanosystem (Samarium Oxide, Neodymium and GQD) was tested at 6 different concentrations of 3.125 ug /mL, 6.25 ug /mL, 12.5 ug /mL, 25 ug /mL, 50 ug /mL, and 100 ug /mL. Positive controls were incorporated into the experiment with the pure compound at the highest concentration tested of 100 ug /mL and at the lowest concentration tested of 3.12 ug /mL. A negative control was added to the experiment containing cells cultured in cell growth medium.

###  Viability assay

 The cells were incubated with the microspheres for 24 hours. After the end of the incubation period, the medium containing the nanosystems was removed, and a 1mg /mL MTT solution was added to each well of the plate. The solution was kept for 2 hours, and after this, a solvent (DMSO) was added to solubilize the formazan crystals for 30 minutes. After the crystals had been completely solubilized, each plate had its absorbance measured on a microplate reader (Multiskan FC; Thermo Fisher Scientific Inc., Waltham, MA, USA) at a wavelength of 450 nm.

## Results and Discussion

**Figure 1:**
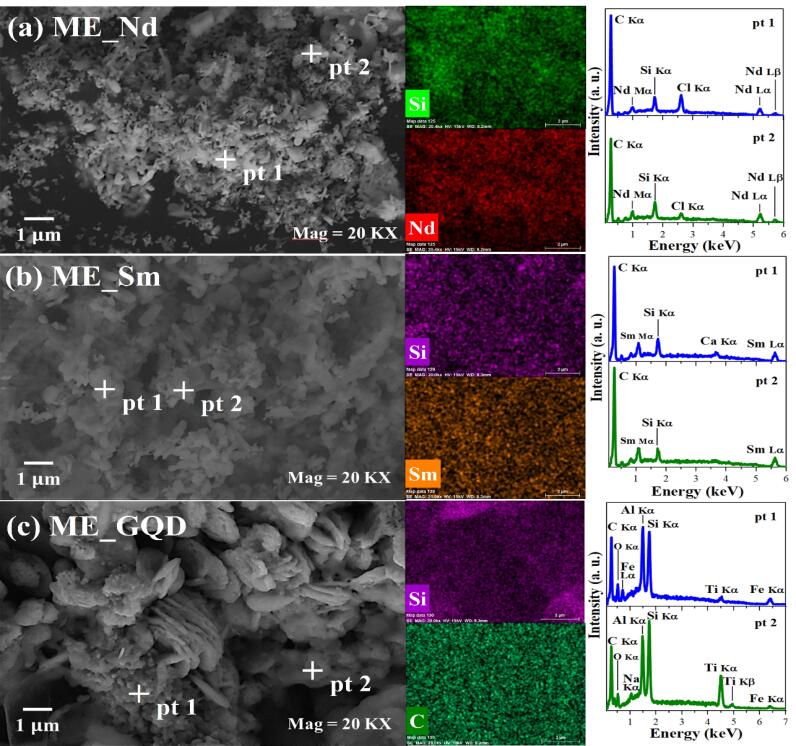
Micrographs, compositional maps, and EDS line spectra were obtained from the points (pt) indicated in the micrographs of the nanoparticles (a) ME_Nd, (b) ME_Sm, and (c) ME_GQD. The identified elements were silicon (Si), neodymium (Nd), samarium (Sm), carbon (C), chlorine (Cl), calcium (Ca), oxygen (O), iron (Fe), aluminum (Al) and titanium (Ti)

**Figure 2:**
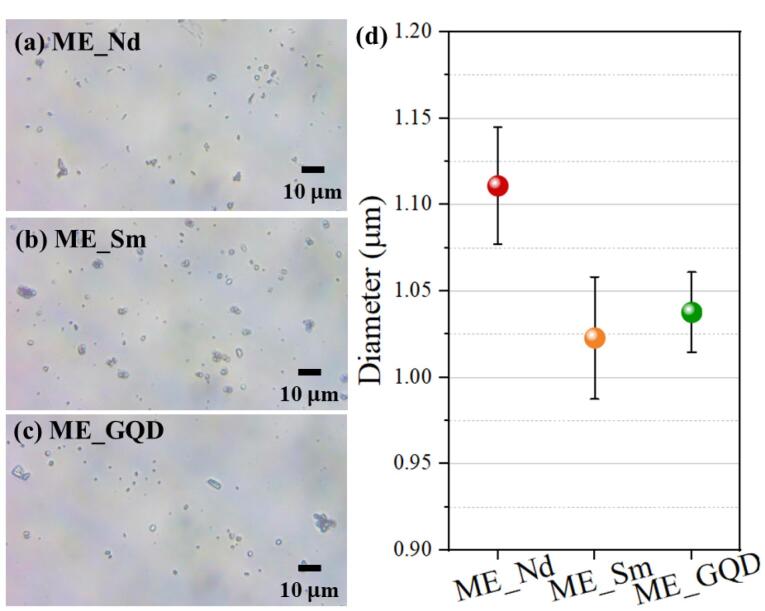
Optical microscopy images of (a) ME_Nd, (b) ME_Sm, and (c) ME_GQD microspheres. (d) Microsphere diameter, where data are presented as mean (sphere) ± standard error (bar) (n = 30)

 The morphology and compositional information of the microspheres obtained by SEM/EDS are presented in Figure 1. The morphology of the ME_Nd and ME_Sm samples (Figure 1a-b) are similar in that they form elongated structures, while the ME_GQD sample (Figure 1c) has greater agglomeration with globular and plate structures. By dispersing the samples in acetone, it was possible to observe the microspheres separately and calculate their diameter with values of 1.11 ± 0.03 µm for ME_Nd, 1.02 ± 0.04 µm for ME_Sm and 1.04 ± 0.02 µm for ME_GQD, according to Figure 2.

 Meanwhile, the compositional analysis, through the maps and EDS spectra, showed the presence of the elements from recycled glass, silicon (Si), carbon (C), chlorine (Cl), calcium (Ca), oxygen (O), iron (Fe), aluminum (Al), and titanium (Ti), as well as the doping elements, Nd, Sm and carbon (C). These elements present uniform distribution, suggesting that the dopants are homogeneous in the material.

 The ME_GQD sample showed morphological and compositional differences from the other samples, with greater particle agglomeration and a high concentration of Al. It is suggested that the greater agglomeration observed in this sample was mainly influenced by Al, given the already observed effect of Al ions in inducing graphene oxide agglomeration compared to Ca and Na ions.^56^

**Figure 3:**
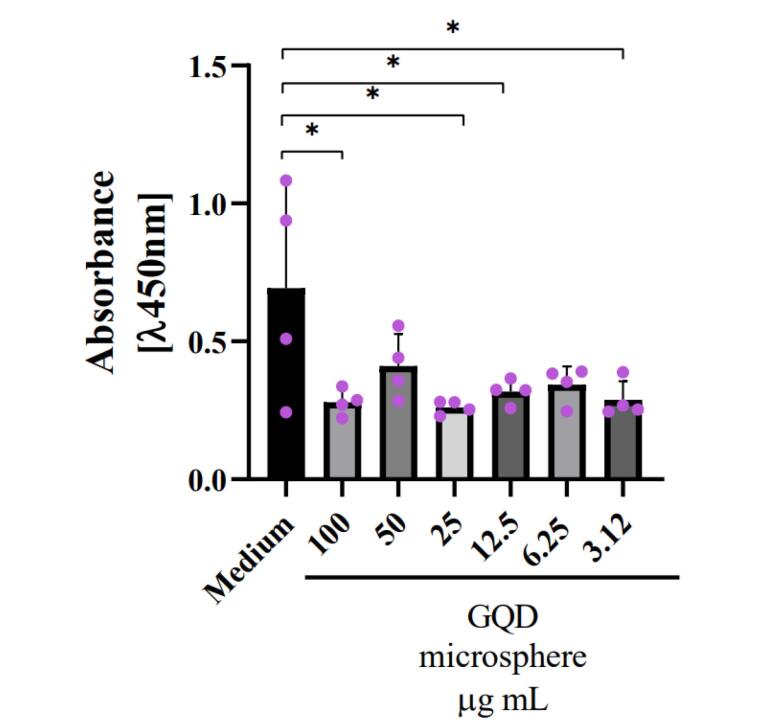
Cell viability assay of prostate tumor cells DU-145 with a GQD microsphere nanosystem.Cell viability was measured after 24 hours of treatment with GQD microspheres at 6 different concentrations of 3.12 ug /mL, 6.25 ug /mL, 12.5 ug /mL, 25 ug /mL, 50 ug /mL, and 100 ug /mL. The X-axis shows the absorbance measured at a wavelength of 450 nm. The Y axis shows the different concentrations tested. The negative control is equivalent to cells in medium only

 The MTT viability assay showed that in all the concentrations of GQD microspheres tested, there was a reduction in cell proliferation for prostate cancer cells, with a focus on the higher concentrations of 100 ug /mL and 25 ug /mL, where this reduction was most evident (*P< 0.05 was considered significant). However, even at lower concentrations, it is possible to observe the impact of this nanosystem on cell viability (Figure 3).

**Figure 4:**
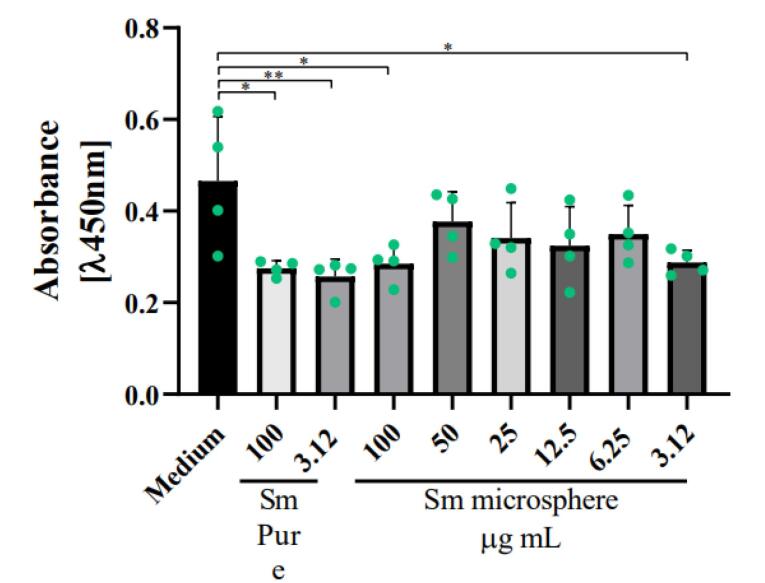
Cell viability assay of breast tumor cells MCF-7 with a Samarium microsphere nanosystem.Cell viability was measured after 24 hours of treatment with Samarium microspheres at 6 concentrations of 3.12 ug /mL, 6.25 ug /mL, 12.5 ug /mL, 25 ug /mL, 50 ug /mL, and 100 ug /mL. The X-axis shows the absorbance measured at a wavelength of 450 nm. The Y axis shows the different concentrations tested. The negative control is equivalent to cells in medium only, and the positive control is with the pure compound at concentrations of 100 ug /mL and 3.12 ug /mL

 Cell viability decreased at the highest and lowest concentrations of 100 ug /mL and 3.12 ug /mL when treated with Samarium microspheres. Similarly, this drop was observed in the positive controls using Samarium in its pure form and at the same concentrations (Figure 4). Despite this, the statistical significance was greater in the controls (**P< 0.01 was considered highly significant) than in the treatment with the nanosystem (*P< 0.05 was considered significant).

**Figure 5:**
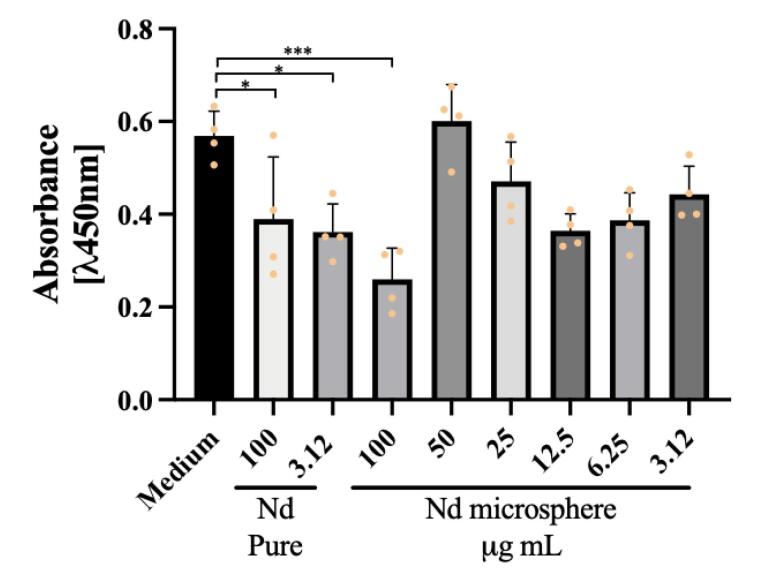
Cell viability assay of breast tumor cell line MCF-7 with a Neodymium microsphere nanosystem.Cell viability was measured after 24 hours of treatment with Neodymium microspheres at 6 different concentrations of 3.12 ug /mL, 6.25 ug /mL, 12.5 ug /mL, 25 ug /mL, 50 ug /mL, and 100 ug /mL. The X-axis shows the absorbance measured at a wavelength of 450 nm. The Y axis shows the different concentrations tested. The negative control is equivalent to cells in medium only, and the positive control is with the pure compound at concentrations of 100 ug /mL and 3.12 ug /mL

**Figure 6:**
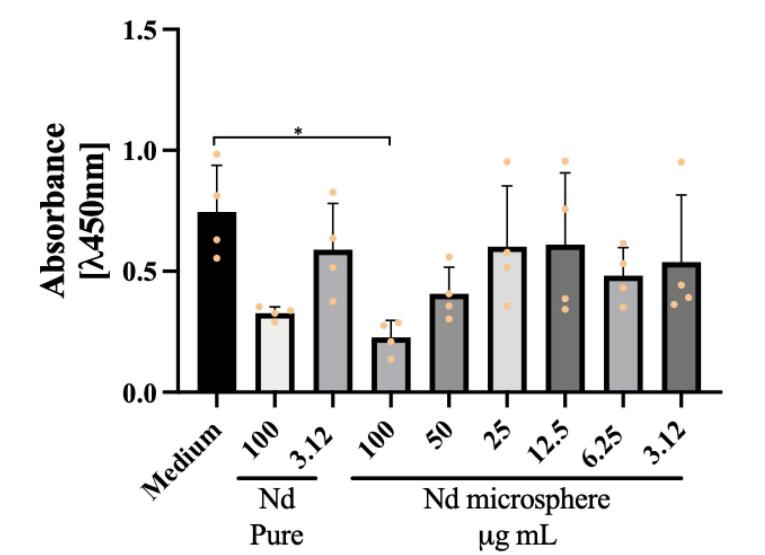
Cell viability assay of prostate tumor cell line DU-145 with a Neodymium microsphere nanosystem.Cell viability was measured after 24 hours of treatment with Neodymium microspheres at 6 different concentrations of 3.12 ug /mL, 6.25 ug /mL, 12.5 ug /mL, 25 ug /mL, 50 ug /mL and 100 ug /mL. The X-axis shows the absorbance measured at a wavelength of 450 nm. The Y axis shows the different concentrations tested. The negative control is equivalent to cells in medium only, and the positive control is with the pure compound at concentrations of 100 ug /mL and 3.12 ug /mL

 As a result, we obtained a decrease in cell proliferation in breast and prostate cancer cells (Figures 5 and 6). This decrease in viability was observed when treated with the highest concentration of the nanosystem, 100 ug /mL, in both tumor cell lines (***P< 0.001 was considered highly significant). In the breast cancer cell lines, we also observed a reduction in cell viability related to treatment with the positive control, pure Neodymium, at the concentrations tested 100 ug /mL and 3.12 ug /mL.

 The production and characterization data showed that the methodology used was able to produce microspheres doped with different types of material efficiently. Also, was possible to obtain glass microspheres with an acceptable range size for biomedical application uniformly doped with the material of interest and with a spherical shape. Although the information about glass microspheres is sparse, the results is confirmed by Li et al^57^ that developed hollow glass microspheres using borosilicate glass composite with different particle size. Due to their micrometer-scale dimensions, irregular morphology, high density, and elevated refractive index, the synthesized glass microspheres are incompatible with colloidal characterization techniques such as DLS and zeta potential analysis. The determination of PDI by laser-based scattering methods is not feasible for solid glass particles of this nature, as they do not remain suspended, do not undergo Brownian motion, and exhibit excessive optical scattering. As such, characterization was instead conducted using SEM and EDS to assess morphology and elemental distribution

 In vitro evaluation revealed a significant reduction in tumor cell viability following treatment with GQDs microspheres. The mechanism behind its role in protecting against cancer progression can be explained by biological pathways such as the activation of the apoptosis pathway or the production of reactive oxygen species (ROS).^58,59^ It has already been shown that graphene and its derivatives can be internalized by cells and interact with various organelles and intracellular molecules, which would alter the cellular microenvironment, triggering inflammatory or apoptotic processes.^60^ In addition, the production of ROS generates cytotoxic effects in cells and mitochondrial disorders, such as a reduction in membrane potential and consequent damage to the membrane.^61^ Is important to notice that IC₅₀ values were not determined, as the observed cytotoxicity did not follow a classic sigmoidal dose–response profile. Only partial viability reduction was observed at certain concentrations, with an absence of a consistent monotonic trend across the tested range. Consequently, curve-fitting models for IC₅₀ derivation could not be applied with sufficient statistical confidence.

 The effect of graphene and its derivatives depends on the characteristics of the particle used, which will vary in size, oxidation, and other physical characteristics. Consequently, this data will change the way in which the particle penetrates biological systems. GQDs are a particle of reduced size which, when combined with glass microspheres, has increased penetration power.^62^ Qin et al described the use of GQDs to test cell viability in macrophages, observing a decrease in the proliferation of these cells at high concentrations of GQDs due to apoptotic events.^63^ These results correlate with our characterization and viability assay findings.

 In addition, it is also described that for tumor cells, including prostate cancer, graphene can alter ATP production, reducing it and subsequently causing impairment of F-actin cytoskeleton assembly. These mechanisms are responsible for preventing cell migration and invasion of these tumor cell lines.^64^

 REEs are widely known and used for their imaging applications in the biomedical field, but they are also being used for biotechnological applications, precisely because they interact with biological molecules, which can be used in anti-tumor therapies.^65^ The results showed a decrease in the cell viability of tumor cell lines in all treatments with microspheres associated with REEs. These nanosystems are possibly modulating DNA damage pathways, ROS production and apoptosis pathways.

 Wei et al have already shown that complexes formed with the element Dysprosium, from the lanthanide family, produce high anti-cancer activity by interfering in the S phase of the cell cycle, causing DNA damage and inducing the apoptosis pathway, preventing tumor progression.^66^

 Furthermore, it has been shown in previous studies with HeLa cells that REEs nanoparticles are also responsible for inhibiting the cyclin dependent kinase 4/cyclin D complex and consequently interrupting tumor cell division.^67^ Nanoparticles of the element Cerium, also from the lanthanide family, induce oxidative stress in tumor cells, activating apoptotic pathways as well as protein kinase (MAPK) signaling pathways, both of which reduce cell viability.^68^

 Rumbo et al observed that Nd nanoparticles caused a dose-dependent production of reactive oxygen species, while Donahue et. al also demonstrated that these same particles have a cytotoxic effect, as observed in several other nanosystems associated with REEs, in correlation with the results found in this study.^69,70^

 It has already been shown that the toxicity of REEs is related to their electronegativity and how these divalent metals have a greater affinity with sites in cells that are in contact with elements such as calcium, zinc and copper, which interferes with cellular homeostasis and promotes dysfunctions that are capable of altering the proliferation of tumor cells when used for this biotechnological purpose.^71^

 Nanomaterials such as glass microspheres associated with REEs or GQDs have great biotechnological potential as tools in anti-tumor therapies, due to their direct actions in various biological processes at the cellular level that promote the destabilization of tumor cells through different signaling pathways. The use of more unusual REEs for this purpose, such as Sm or Nd, should be further explored, given that, like cerium or gadolinium, they are also successful in decreasing cell viability, as can be seen in this study. Is important to notice that the hybrid glass microsphere system exhibits enhanced structural stability, limiting the premature degradation and systemic leakage often observed in polymeric and lipid-based carriers. The integration of rare earth dopants and GQDs within the glass matrix enables localized functionalization while maintaining material integrity. This design not only enhances safety and control but also introduces potential theranostic functionality. Furthermore, the use of recycled glass substrates supports a sustainable, cost-effective production route.

## Conclusion

 The study concludes that glass microspheres doped with REEs, Sm and Nd, along with GQDs, show significant potential as therapeutic agents in cancer treatment. These nanosystems demonstrated a considerable reduction in cell viability across both breast (MCF-7) and prostate (DU-145) cancer cell lines, primarily through mechanisms involving apoptosis and ROS production. The efficacy of the GQD microspheres, in particular, highlights their promise for targeted cancer therapies due to their biocompatibility and low cytotoxicity. Similarly, the REEs Sm and Nd proved effective in reducing cancer cell survival, with notable activity in disrupting cellular processes critical for tumor growth and proliferation. The physicochemical stability of the microspheres in biological environments is an important consideration. Given the silica-based glassy nature of the matrix, these systems are expected to exhibit low solubility and high stability under physiological conditions. While no degradation or structural alterations were observed during the 24-hour in vitro assays, future studies will assess their behavior in biological fluids over extended periods and under dynamic conditions to better simulate in vivo environments. This research underscores the importance of further exploring the unique properties of these materials in oncology, particularly for developing novel, less toxic cancer treatments. The findings suggest that these doped microspheres could play a pivotal role in advancing nanomedicine, offering a new avenue for combating resistant and metastatic cancers.

## Competing Interests

 The authors state that they have no conflict of interest.

## Data Availability Statement

 All data will be available under request.

## Ethical Approval

 All procedures were performed in accordance with the Health Guide for the Care and Use of Experimental Animals (CEUA) and approved by the Rio de Janeiro State University Committee (protocol 8059100220/2021), which is in line with the NIH Guidelines for the Care and Use of Laboratory Animals.

## References

[1] Righini GC (2018). Glassy microspheres for energy applications. Micromachines (Basel).

[2] Righini GC. Glass Micro- and Nanospheres: Physics and Applications. 1st ed. New York: Jenny Stanford Publishing; 2019.

[3] Yu H, Huang Q, Zhao J (2014). Fabrication of an optical fiber micro-sphere with a diameter of several tens of micrometers. Materials (Basel).

[4] Elliott GR, Hewak DW, Murugan GS, Wilkinson JS (2007). Chalcogenide glass microspheres; their production, characterization and potential. Opt Express.

[5] Chiesa C, Mira M, Maccauro M, Spreafico C, Romito R, Morosi C (2015). Radioembolization of hepatocarcinoma with 90Y glass microspheres: development of an individualized treatment planning strategy based on dosimetry and radiobiology. Eur J Nucl Med Mol Imaging.

[6] Wright CL, Zhang J, Tweedle MF, Knopp MV, Hall NC (2015). Theranostic imaging of yttrium-90. Biomed Res Int.

[7] Poorbaygi H, Aghamiri SM, Sheibani S, Kamali-Asl A, Mohagheghpoor E (2011). Production of glass microspheres comprising 90Y and 177Lu for treating of hepatic tumors with SPECT imaging capabilities. Appl RadiatIsot.

[8] Gupta S, Majumdar S, Krishnamurthy S (2021). Bioactive glass: a multifunctional delivery system. J Control Release.

[9] Sharifi E, Bigham A, Yousefiasl S, Trovato M, Ghomi M, Esmaeili Y (2022). Mesoporous bioactive glasses in cancer diagnosis and therapy: stimuli-responsive, toxicity, immunogenicity, and clinical translation. Adv Sci (Weinh).

[10] Gupta T, Virmani S, Neidt TM, Szolc-Kowalska B, Sato KT, Ryu RK (2008). MR tracking of iron-labeled glass radioembolization microspheres during transcatheter delivery to rabbit VX2 liver tumors: feasibility study. Radiology.

[11] Yang J, Kang H, Shi C, Hu X, Moon J (2024). Tomographic analysis of segregation behavior of hollow glass microspheres in lightweight cementitious composites. CemConcr Compos.

[12] Gerhardt LC, Boccaccini AR (2010). Bioactive glass and glass-ceramic scaffolds for bone tissue engineering. Materials (Basel).

[13] Zheng B, Fan J, Chen B, Qin X, Wang J, Wang F (2022). Rare-earth doping in nanostructured inorganic materials. Chem Rev.

[14] Royal Society of Chemistry. Samarium - Element Information, Properties and Uses. Available from: https://www.rsc.org/periodic-table/element/62/samarium. Accessed December 10, 2024.

[15] Feng Y, Xiao Q, Zhang Y, Li F, Li Y, Li C (2017). Neodymium-doped NaHoF4 nanoparticles as near-infrared luminescent/T2-weighted MR dual-modal imaging agents in vivo. J Mater Chem B.

[16] Sheikh Mohd Ghazali SA, Fatimah I, Zamil ZN, Zulkifli NN, Adam N (2023). Graphene quantum dots: a comprehensive overview. Open Chem.

[17] Kadyan P, Malik R, Bhatia S, Al Harrasi A, Mohan S, Yadav M (2023). Comprehensive review on synthesis, applications, and challenges of graphene quantum dots (GQDs). J Nanomater.

[18] Magne TM, de Oliveira Vieira T, Alencar LM, Junior FF, Gemini-Piperni S, Carneiro SV (2022). Graphene and its derivatives: understanding the main chemical and medicinal chemistry roles for biomedical applications. J Nanostructure Chem.

[19] da Cunha Goldstein A, Araujo-Lima CF, da Silva Fernandes A, Santos-Oliveira R, Felzenszwalb I (2023). In vitro genotoxicity assessment of graphene quantum dots nanoparticles: a metabolism-dependent response. Mutat Res Genet Toxicol Environ Mutagen.

[20] Antoine C, Sahylí Ortega Pijeira M, Ricci-Junior E, Magalhães Rebelo Alencar L, Santos-Oliveira R (2022). Graphene quantum dots as bimodal imaging agent for X-ray and computed tomography. Eur J Pharm Biopharm.

[21] Li Y, Dong H, Tao Q, Ye C, Yu M, Li J (2020). Enhancing the magnetic relaxivity of MRI contrast agents via the localized superacid microenvironment of graphene quantum dots. Biomaterials.

[22] Bray F, Laversanne M, Sung H, Ferlay J, Siegel RL, Soerjomataram I (2024). Global cancer statistics 2022: GLOBOCAN estimates of incidence and mortality worldwide for 36 cancers in 185 countries. CA Cancer J Clin.

[23] Paredes LP, da Silva Brandao Rodrigues M, Santos-Oliveira R (2024). Deciphering trends in cancer mortality: a comprehensive analysis of Brazilian data from 1979 to 2021 with emphasis on breast and prostate cancers. World J Oncol.

[24] Arnold M, Morgan E, Rumgay H, Mafra A, Singh D, Laversanne M (2022). Current and future burden of breast cancer: global statistics for 2020 and 2040. Breast.

[25] Łukasiewicz S, Czeczelewski M, Forma A, Baj J, Sitarz R, Stanisławek A (2021). Breast Cancer-epidemiology, risk factors, classification, prognostic markers, and current treatment strategies-an updated review. Cancers (Basel).

[26] Arzanova E, Mayrovitz HN. The Epidemiology of Breast Cancer. Brisbane, AU: Exon Publications; 2022. 36122161

[27] Campbell JD, Ramsey SD (2009). The costs of treating breast cancer in the US: a synthesis of published evidence. Pharmacoeconomics.

[28] Khushalani JS, Trogdon JG, Ekwueme DU, Yabroff KR (2019). Economics of public health programs for underserved populations: a review of economic analysis of the National Breast and Cervical Cancer Early Detection Program. Cancer Causes Control.

[29] James ND, Tannock I, N’Dow J, Feng F, Gillessen S, Ali SA (2024). The Lancet Commission on prostate cancer: planning for the surge in cases. Lancet.

[30] Porcacchia AS, Pires GN, Ortiz V, Andersen ML, Tufik S (2022). Prostate cancer mortality and costs of prostate surgical procedures in the Brazilian public health system. Int Braz J Urol.

[31] Olsen TA, Filson CP, Richards TB, Ekwueme DU, Howard DH (2023). The cost of metastatic prostate cancer in the United States. UrolPract.

[32] Cantarero-Prieto D, Lera J, Lanza-Leon P, Barreda-Gutierrez M, Guillem-Porta V, Castelo-Branco L (2022). The economic burden of localized prostate cancer and insights derived from cost-effectiveness studies of the different treatments. Cancers (Basel).

[33] World Health Organization (WHO). Global Cancer Burden Growing, Amidst Mounting Need for Services. WHO; 2024. Available from: https://www.who.int/news/item/01-02-2024-global-cancer-burden-growing--amidst-mounting-need-for-services. Accessed December 10, 2024.

[34] Piñeros M, Laversanne M, Barrios E, de Camargo Cancela M, de Vries E, Pardo C (2022). An updated profile of the cancer burden, patterns and trends in Latin America and the Caribbean. Lancet Reg Health Am.

[35] Hanna TP, King WD, Thibodeau S, Jalink M, Paulin GA, Harvey-Jones E (2020). Mortality due to cancer treatment delay: systematic review and meta-analysis. BMJ.

[36] Yeo HY, Liew AC, Chan SJ, Anwar M, Han CH, Marra CA (2023). Understanding patient preferences regarding the important determinants of breast cancer treatment: a narrative scoping review. Patient Prefer Adherence.

[37] Passaro A, Al Bakir M, Hamilton EG, Diehn M, André F, Roy-Chowdhuri S (2024). Cancer biomarkers: emerging trends and clinical implications for personalized treatment. Cell.

[38] Wang Y, Minden A (2022). Current molecular combination therapies used for the treatment of breast cancer. Int J Mol Sci.

[39] Gonzalez-Angulo AM, Morales-Vasquez F, Hortobagyi GN (2007). Overview of resistance to systemic therapy in patients with breast cancer. Adv Exp Med Biol.

[40] Rej RK, Roy J, Allu SR (2024). Therapies for the treatment of advanced/metastatic estrogen receptor-positive breast cancer: current situation and future directions. Cancers (Basel).

[41] Reinert T, de Paula B, Shafaee MN, Souza PH, Ellis MJ, Bines J (2018). Endocrine therapy for ER-positive/HER2-negative metastatic breast cancer. Chin Clin Oncol.

[42] Mosleh-Shirazi S, Abbasi M, Moaddeli MR, Vaez A, Shafiee M, Kasaee SR (2022). Nanotechnology advances in the detection and treatment of cancer: an overview. Nanotheranostics.

[43] Mugundhan SL, Mohan M (2024). Nanoscale strides: exploring innovative therapies for breast cancer treatment. RSC Adv.

[44] Tiwari H, Gupta P, Verma A, Singh S, Kumar R, Gautam HK (2024). Advancing era and rising concerns in nanotechnology-based cancer treatment. ACS Chem Health Saf.

[45] Sakore P, Bhattacharya S, Belemkar S, Prajapati BG, Elossaily GM (2024). The theranostic potential of green nanotechnology-enabled gold nanoparticles in cancer: a paradigm shift on diagnosis and treatment approaches. Results Chem.

[46] Elumalai K, Srinivasan S, Shanmugam A (2024). Review of the efficacy of nanoparticle-based drug delivery systems for cancer treatment. Biomed Technol.

[47] Giri PM, Banerjee A, Layek B (2023). A recent review on cancer nanomedicine. Cancers (Basel).

[48] Ghazal H, Waqar A, Yaseen F, Shahid M, Sultana M, Tariq M (2024). Role of nanoparticles in enhancing chemotherapy efficacy for cancer treatment. Next Mater.

[49] Bajracharya R, Song JG, Patil BR, Lee SH, Noh HM, Kim DH (2022). Functional ligands for improving anticancer drug therapy: current status and applications to drug delivery systems. Drug Deliv.

[50] Xia Y, Sun M, Huang H, Jin WL (2024). Drug repurposing for cancer therapy. Signal Transduct Target Ther.

[51] Talib WH, Alsayed AR, Barakat M, Abu-Taha MI, Mahmod AI (2021). Targeting drug chemo-resistance in cancer using natural products. Biomedicines.

[52] Krzyszczyk P, Acevedo A, Davidoff EJ, Timmins LM, Marrero-Berrios I, Patel M (2018). The growing role of precision and personalized medicine for cancer treatment. Technology (Singap World Sci).

[53] Edsjö A, Holmquist L, Geoerger B, Nowak F, Gomon G, Alix-Panabières C (2023). Precision cancer medicine: concepts, current practice, and future developments. J Intern Med.

[54] Subhan MA, Parveen F, Shah H, Yalamarty SS, Ataide JA, Torchilin VP (2023). Recent advances with precision medicine treatment for breast cancer including triple-negative sub-type. Cancers (Basel).

[55] de Menezes FD, Dos Reis SR, Pinto SR, Portilho FL, do Vale Chaves EM, Helal-Neto E (2019). Graphene quantum dots unraveling: green synthesis, characterization, radiolabeling with 99mTc, in vivo behavior and mutagenicity. Mater Sci Eng C Mater Biol Appl.

[56] Chow MK, Jee CE, Yeap SP (2022). Qualitative and quantitative determination of critical coagulation concentration for pristine graphene oxide in various ionic compounds. Results Eng.

[57] Ren S, Li X, Zhang X, Xu X, Dong X, Liu J (2017). Mechanical properties and high-temperature resistance of the hollow glass microspheres/borosilicate glass composite with different particle size. J Alloys Compd.

[58] Wang D, Zhu L, Chen JF, Dai L (2015). Can graphene quantum dots cause DNA damage in cells?. Nanoscale.

[59] Wang X, Hu C, Gu Z, Dai L (2021). Understanding of catalytic ROS generation from defect-rich graphene quantum-dots for therapeutic effects in tumor microenvironment. J Nanobiotechnology.

[60] Zhang B, Wei P, Zhou Z, Wei T (2016). Interactions of graphene with mammalian cells: molecular mechanisms and biomedical insights. Adv Drug Deliv Rev.

[61] Ou L, Song B, Liang H, Liu J, Feng X, Deng B (2016). Toxicity of graphene-family nanoparticles: a general review of the origins and mechanisms. Part FibreToxicol.

[62] Magne TM, de Oliveira Vieira T, Costa B, Alencar LM, Ricci-Junior E, Hu R (2021). Factors affecting the biological response of graphene. Colloids Surf B Biointerfaces.

[63] Qin Y, Zhou ZW, Pan ST, He ZX, Zhang X, Qiu JX (2015). Graphene quantum dots induce apoptosis, autophagy, and inflammatory response via p38 mitogen-activated protein kinase and nuclear factor-κB mediated signaling pathways in activated THP-1 macrophages. Toxicology.

[64] Zhou H, Zhang B, Zheng J, Yu M, Zhou T, Zhao K (2014). The inhibition of migration and invasion of cancer cells by graphene via the impairment of mitochondrial respiration. Biomaterials.

[65] Rocha RA, Alexandrov K, Scott C (2024). Rare earth elements in biology: from biochemical curiosity to solutions for extractive industries. MicrobBiotechnol.

[66] Liu YC, Chen ZF, Song XY, Peng Y, Qin QP, Liang H (2013). Synthesis, crystal structure, cytotoxicity and DNA interaction of 5,7-dibromo-8-quinolinolato-lanthanides. Eur J Med Chem.

[67] Chan CF, Tsang MK, Li H, Lan R, Chadbourne FL, Chan WL (2014). Bifunctional up-converting lanthanide nanoparticles for selective in vitro imaging and inhibition of cyclin D as anti-cancer agents. J Mater Chem B.

[68] Cheng G, Guo W, Han L, Chen E, Kong L, Wang L (2013). Cerium oxide nanoparticles induce cytotoxicity in human hepatoma SMMC-7721 cells via oxidative stress and the activation of MAPK signaling pathways. Toxicol In Vitro.

[69] Donohue VE, McDonald F, Evans R (1995). In vitro cytotoxicity testing of neodymium-iron-boron magnets. J Appl Biomater.

[70] Rumbo C, Espina CC, Popov VV, Skokov K, Tamayo-Ramos JA (2021). Toxicological evaluation of MnAl based permanent magnets using different in vitro models. Chemosphere.

[71] Dubé M, Auclair J, Hanana H, Turcotte P, Gagnon C, Gagné F (2019). Gene expression changes and toxicity of selected rare earth elements in rainbow trout juveniles. Comp BiochemPhysiol C ToxicolPharmacol.

